# Alcohol as a Modifiable Risk Factor for Alzheimer’s Disease—Evidence from Experimental Studies

**DOI:** 10.3390/ijms24119492

**Published:** 2023-05-30

**Authors:** Devaraj V. Chandrashekar, Ross A. Steinberg, Derick Han, Rachita K. Sumbria

**Affiliations:** 1 Department of Biomedical and Pharmaceutical Sciences, School of Pharmacy, Chapman University, Irvine, CA 92618, USA; chandnrashekar@chapman.edu; 2 School of Pharmacy and Health Sciences, Keck Graduate Institute, Claremont, CA 91711, USA; rsteinberg16@kgi.edu (R.A.S.); derick_han@kgi.edu (D.H.); 3 Department of Neurology, University of California, Irvine, CA 92697, USA

**Keywords:** Alzheimer’s disease, β-amyloid, alcohol, animal models, cell culture, liver injury, liver steatosis

## Abstract

Alzheimer’s disease (AD) is a progressive neurodegenerative disease characterized by cognitive impairment and memory loss. Epidemiological evidence suggests that heavy alcohol consumption aggravates AD pathology, whereas low alcohol intake may be protective. However, these observations have been inconsistent, and because of methodological discrepancies, the findings remain controversial. Alcohol-feeding studies in AD mice support the notion that high alcohol intake promotes AD, while also hinting that low alcohol doses may be protective against AD. Chronic alcohol feeding to AD mice that delivers alcohol doses sufficient to cause liver injury largely promotes and accelerates AD pathology. The mechanisms by which alcohol can modulate cerebral AD pathology include Toll-like receptors, protein kinase-B (Akt)/mammalian target of rapamycin (mTOR) pathway, cyclic adenosine monophosphate (cAMP) response element-binding protein phosphorylation pathway, glycogen synthase kinase 3-β, cyclin-dependent kinase-5, insulin-like growth factor type-1 receptor, modulation of β-amyloid (Aβ) synthesis and clearance, microglial mediated, and brain endothelial alterations. Besides these brain-centric pathways, alcohol-mediated liver injury may significantly affect brain Aβ levels through alterations in the peripheral-to-central Aβ homeostasis. This article reviews published experimental studies (cell culture and AD rodent models) to summarize the scientific evidence and probable mechanisms (both cerebral and hepatic) by which alcohol promotes or protects against AD progression.

## 1. Introduction

Alzheimer’s disease (AD) is histologically characterized by the deposition of β-amyloid (Aβ) plaques and hyperphosphorylated tau protein-rich intraneuronal neurofibrillary tangles (NFT) [1]. AD causes gradual deterioration in cognitive function, worsening behavioral manifestations, and is the most common cause of dementia globally. Epidemiological data show that the number of AD patients is expected to increase to 152 million by 2050, placing it among the leading causes of death worldwide [1].

Many potentially modifiable risk factors may contribute to dementia, including alcohol drinking, smoking, poor diet, physical inactivity, cognitive inactivity, depression, and low educational attainment [2,3]. Non-modifiable risk factors for AD include family history, aging, and genetic factors, including the epsilon 4 allele of the apolipoprotein E (APOE4) gene for late-onset AD and amyloid precursor protein (APP) and presenilin (PSEN) single-mutations for early onset AD [3].

It is estimated that around 107 million people (approximately 1% of the global population) suffer from alcohol use disorder, of which 70% are male and 30% are female, with a respective death rate of 7.7% and 2.6% [4]. Alcohol consumption, whether acute or chronic, is a significant health and economic problem and has been suggested to be a modifiable risk factor for various diseases, including AD. The effects of alcohol intake on AD pathology and progression are debated, as it shows both protective and aggravating effects. These varying effects are likely associated with the dose, drinking pattern, genetics, fed or fasting state of the individual, and the duration of alcohol intake [5,6]. In this review, we have summarized in vivo and cell culture studies that elucidate the effects of alcohol on AD pathology, whether protective or detrimental, and the underlying mechanisms, both cerebral and peripheral, modulated by alcohol in potentiating or attenuating AD pathology.

## 2. Alcohol Intake and AD: Evidence from Epidemiological Studies

The available epidemiological data regarding the contribution of alcohol to cognitive impairment and AD-type neurodegeneration have been controversial due to variability and inconsistencies in both alcohol and AD measurement parameters [5]. However, a pattern has emerged, with some epidemiological studies suggesting that low alcohol intake reduces the prevalence of AD [7,8,9], whereas chronic heavy alcohol intake promotes dementia and leads to the deterioration of cognitive and executive function [8]. For example, a study of a Chinese population showed that light drinking (i.e., weekly alcohol intake between 168 to 400 g for men and 112 to 280 g for women) reduced the risk of cognitive impairment, while heavy drinking (i.e., weekly alcohol intake > 400 g for men and >280 g for women) increased the risk of cognitive impairment as assessed by the Mini-Mental State Examination (MMSE), an index to measure cognitive status [8]. Similarly, a cohort study in the Korean population showed that sustained mild to moderate alcohol drinking (between 15 to 29.9 g per day) reduced the risk of dementia by 17 to 21%, while sustained heavy alcohol intake (≥30 g per day) increased the risk of dementia by 8% [9]. Further, in a prospective cohort study of older adults, low-quantity drinking (1.0 drink per week) was associated with a lower dementia risk in participants without mild cognitive impairment (MCI). In contrast, high-quantity drinking (>14 drinks per week) was associated with severe cognitive decline in participants with MCI [10]. Overall, protective effects of light-to-moderate alcohol intake have been observed in at least seven studies, whereas other studies report no beneficial and even detrimental effects of light-to-moderate alcohol intake on AD and related dementia [5]. The probable mechanism involved in the protective effect of low alcohol intake on cognitive impairment is the acetylcholine release in the hippocampal region, which enhances learning and memory [11]. Another possible mechanism could be reduced cardiovascular risk factors such as decreased platelet aggregation or decreased serum lipid profile [11]. However, whether low alcohol consumption is protective against AD remains controversial [5] due to other confounding factors such as the impact of social interaction with alcohol drinking and differences in alcohol metabolism, and the World Health Organization does not recommend alcohol consumption for protection against AD.

In contrast, the literature appears to be more convergent with respect to the effect of heavy alcohol consumption on the risk of AD and other dementias. Heavy alcohol consumption has been linked to an increased risk of dementia and AD in numerous studies [6,9,12,13], although this has not been universally observed [12,14]. A systematic scoping review of epidemiological data on alcohol and AD concluded that heavy alcohol use was associated with changes in brain structure, cognitive impairment, and an increased risk of all types of dementia [12]. It has been suggested that heavy alcohol consumption (defined as >14 drink units/week) increases the risk of AD and related dementias [5]. Thus, heavy alcohol consumption is often categorized as a modifiable risk factor for AD [5,12,15]. Overall, despite many studies examining the effects of alcohol on AD, controversies remain, given the heterogeneity in the studies with respect to the alcohol consumption pattern, age of the subjects, type of alcoholic beverages consumed, duration of alcohol intake, and underlying comorbidities. Whether these results show causality with respect to the effects of alcohol in potentiating or alleviating the risk of AD can be more effectively addressed with randomized controlled trials, which are faced with ethical concerns due to the addictive and toxic effects of alcohol. In this regard, studies in rodent models and cell culture systems offer a controlled environment to isolate the effects of alcohol on AD, and have provided valuable mechanistic insights regarding the pathways modulated by alcohol in AD. Since there have been many extensive reviews of the epidemiological data regarding the impact of alcohol on AD [5,12,16,17], our review will instead focus on the potential mechanistic link between alcohol and AD based on animal and cell models.

## 3. Impact of Alcohol Intake on AD: Evidence from Rodent Models

### 3.1. Alcohol-Feeding Models in AD Mice and Rats

Since the effect of alcohol on AD pathology may depend on alcohol dosing, it is important to first review the models of alcohol feeding to animals and alcohol dosing, as well as their limitations. Overall, it has historically been challenging to mimic very heavy chronic alcohol drinkers in rodent models because most animals, including mice and rats, have a natural aversion to alcohol and thus will not naturally consume alcohol in large amounts [18]. Consequently, most studies have mixed alcohol into a liquid diet (Lieber DeCarli diet) or in water to encourage rats and mice to consume alcohol [19]. However, even under such conditions, rats and mice will limit the consumption of the liquid diet or water to avoid excessive alcohol intake. The following are the most common models used to deliver alcohol to mice and rats.

#### 3.1.1. Chronic Alcohol Feeding through Mixing Alcohol with a Liquid Diet (Lieber DeCarli Diet)

This model mixes alcohol (generally 36% of calories) in a liquid diet to encourage animals to consume alcohol, with feeding usually lasting for 4–8 weeks. However, because of the animal’s aversion to alcohol, they will consume less liquid diet containing alcohol than animals given a control liquid diet. Consequently, a pair matching of a control diet consumption must be performed in this model. It has been difficult to extrapolate alcohol dosing in mice and rats to humans, but based on liver pathology, the Lieber Decarli diet in mice has been generally considered a model of mild alcoholic liver disease (ALD), which is associated with moderate chronic alcoholic drinking [18,20]. Alcohol feeding through a liquid diet to mice and rats primarily produces liver pathology—liver steatosis with mild elevation in alanine transaminase (ALT), a marker of liver injury—generally observed with moderate alcohol drinking in humans. The more advanced alcoholic liver disease symptoms observed in 20–40% of heavy drinkers, including steatohepatitis (fatty liver plus inflammation) and cirrhosis (significant hepatocyte death and fibrosis), do not occur with chronic alcohol intake through diet in mice and rats [20]. Notably, mice generally develop greater alcoholic liver injury than rats, possibly due to differences in betaine homocysteine methyltransferase expression [21].

#### 3.1.2. Chronic Alcohol Feeding through Mixing Alcohol in Drinking Water

Alcohol can be mixed into drinking water (the sole source of water), while allowing animals to receive standard chow ad libitum. Alcohol content of 10–20% in drinking water (*v*/*v*) is standard, but up to 40% can be achieved if the alcohol content is gradually increased. To further encourage alcohol consumption in drinking water, many variations have been developed, including adding saccharine as a sweetener to the water, alternating pure water with alcohol plus water throughout the week (two-bottle choice drinking paradigm), and providing alcohol in water 3 h prior to the dark cycle (the drinking in the dark model) [22,23]. The latter two variations cause alcohol intake in binges and are sometimes considered alcohol binge models. The extent of liver injury caused by spiking alcohol in drinking water will depend on the duration and alcohol content; however, if the two are sufficient, this model can cause liver steatosis and elevated ALT levels similar to the Lieber Decarli diet described above [23,24]. Additionally, similar to the Lieber Decarli diet, steatohepatitis and liver fibrosis cannot be achieved with this model alone, again suggesting that this model mimics moderate alcohol drinking [23]. An advantage of this alcohol delivery method is that it can be performed long-term for months, but a disadvantage is that because the animals obtain calories from alcohol in drinking water, the nutritional status of alcohol versus control animals may vary.

#### 3.1.3. Chronic Alcohol Binges

Since many alcoholics are known to binge drink, many researchers mimic binge drinking by providing one high alcohol dose daily through gavage or through intraperitoneal injection (i.p.). The liver injury caused by binge drinking will vary by dose and duration of alcohol delivery, but chronic dosing for an extended period can cause liver steatosis and increase ALT, as seen in the two models described above. However, similar to the other models described thus far, daily binge delivery of alcohol cannot cause steatohepatitis and liver fibrosis in animals [20,23].

#### 3.1.4. Intragastric Alcohol Feeding

To mimic chronic heavy alcohol intake in humans, the intragastric alcohol-feeding model was developed [25]. This model uses a catheter that is surgically implanted into the stomach to force-feed alcohol with a liquid diet into mice and rats for 4–6 weeks [26,27]. Because of the large amounts of alcohol force-fed to mice and rats, the intragastric feeding model produces the highest liver steatosis and ALT release seen in any alcohol-feeding model, as well as some inflammation and slight fibrosis [18]. Although this model produces the most severe liver injury of all alcohol-feeding models because of its high alcohol delivery, it still does not cause significant hepatic inflammation and fibrosis to the extent seen in many heavy-drinking alcoholic patients [18,20]. Due to the difficulty of the surgical implantation and its resultant elevated mortality rate, this model has not been attempted in AD mice to our knowledge. However, we recently utilized intragastric alcohol feeding for wild-type mice (C57BL/6J mice) and observed the loss of microglia cells in the cerebral cortex and the modulation of hepatic APP and low-density lipoprotein-related protein 1 (LRP1), which will be discussed later in the review [28].

#### 3.1.5. National Institute on Alcohol Abuse and Alcoholism Chronic and Binge Alcohol Feeding

The National Institute on Alcohol Abuse and Alcoholism (NIAAA) chronic binge model mixes the oral liquid diet feeding of alcohol with a large alcohol binge at the end. The combination of chronic alcohol feeding with a binge has been shown to cause higher steatosis and ALT release than the traditional delivery of alcohol in drinking water or in a liquid diet [20]. The NIAAA binge model is associated with higher mortality rates and, to our knowledge, has also not been attempted in AD mice.

In summary, it has been challenging to feed alcohol to animals because of their aversion to alcohol. Most alcohol-feeding models that have been performed in AD mice, which will be discussed in the next section, generate liver pathology associated with chronic moderate drinking rather than heavy drinking.

### 3.2. Animal Studies Showing an Increase in AD Pathology with Alcohol

The most compelling evidence for the effect of alcohol on AD has come from chronic alcohol feeding to mice that harbor mutations observed in patients with early onset familial AD. The summary and mechanistic insights obtained from these alcohol-feeding studies to AD mice are highlighted below and in Table 1.

#### 3.2.1. Chronic Alcohol Feeding to Mature-Adult 3xTg-AD Mice

In three-month-old male and female 3xTg-AD mice that harbor mutations in the APP, PSEN1, and tau genes, chronic voluntary alcohol consumption (25% *w*/*v* alcohol + 0.1% saccharin *w*/*v* in water) for four months increased the Aβ (42/40) ratio and total tau protein in the lateral entorhinal cortex (LEC) and prefrontal cortex (PFC), which is consistent with enhanced AD-related pathological changes. Alcohol upregulated the immunoreactivity of phosphorylated tau (Ser199/Ser202) in the hippocampal region of the AD mice. Alcohol did not affect normal locomotor activity but altered the spatial memory and cognitive functions in the 3xTg-AD mice. Alcohol intake also dysregulated the emotional response of the AD mice. Thus, alcohol consumption appears to augment the onset of PFC-linked AD pathology in mature-adult 3xTg-AD mice [29].

**Table 1:** List of animal studies reporting the effect of alcohol in AD rodent models.

Strain	Model	Behavioral Testing	Biomarkers Measured	Outcome	Ref. #
3xTg and WT mice Age (start): ~3 months Age (end): ~7 months	Oral sweetened alcohol for 4 months (25% *w*/*v* alcohol + saccharin 0.1% *w*/*v*)	Open field test to assess locomotion. Rotarod to assess motor coordination and balance. Morris water maze test to evaluate spatial memory. Pre-pulse inhibition to assess sensorimotor gating and startle response. Fear conditioning to assess emotional processing and learning and memory.	Aβ: Aβ40, Aβ42, Tau: p-tau Thr181, Ser-199/Ser202, Akt/mTOR phospho-proteins: p70S6K (Thr412), IRS1 (Ser636), IGF1R (Tyr1135 /Tyr1136), PTEN (Ser380), ERK/MAPK ½ (Thr185/Tyr187), RPS6 (Ser235 /Ser236) IR (Tyr1162 /Tyr1163), mTOR (Ser2448), GSK3α (Ser21), GSK3β (Ser9)	Alcohol treatment did not change motor ability, but impaired spatial memory and increased fear response in 3xTg mice. Alcohol also increased the Aβ (42/40) ratio and total tau in LEC and PFC, upregulated pTau (Ser199/Ser202), and dysregulated Akt/mTOR phosphoproteins in the 3xTg mice. **Conclusion:** Chronic alcohol intake aggravates amyloid and tau pathology in the 3xTg mice.	[29]
APP23/PS45 mice Age (start): ~2 months Age (end): ~3 months	Oral sweetened alcohol for 4 weeks (20% *v*/*v* alcohol + saccharin 0.07% *w*/*v*)	Morris water maze test to assess spatial memory.	Aβ40, Aβ42, APP, APP-CTFs, and BACE-1	Chronic alcohol intake impaired spatial memory and increased Aβ40, Aβ42, APP, BACE-1, and APP-CTF β-fragments (C-99 and C-89) in APP23/PS45 mice. **Conclusion:** Alcohol exposure promotes APP processing and aggravates AD pathology.	[30]
APPswe/PS1dE9 mice Age (start): ~5.5 months Age (end): ~8 months	20% alcohol in drinking water for 12 h/day for 4 consecutive days per week for 10 weeks	Open field test and light/dark assay to assess locomotion and anxiety-like behavior. Marble burying to assess repetitive and compulsive behavior. Object location memory task to assess spatial memory. Nest building to assess self-care.	Aβ40 and Aβ42 mRNA levels of NMDA and GABAA receptors	Chronic alcohol treatment increased locomotor activity and modulated mRNA levels of NMDA (increases) and GABAA (decreases) receptors in APP/PS1 mice brains. Alcohol elevated the hippocampal interstitial fluid Aβ40 levels with no changes in the Aβ42 levels. **Conclusion:** Chronic alcohol consumption aggravates AD-related pathology in the APPswe/PS1dE9 mice.	[31]
APPswe/PS1dE9 mice Age (start): ~4 months Age (end): ~6 months	Alcohol dosed orally at 10 mL/kg daily for 2 months	Passive avoidance test to assess learning and memory. Morris water maze test to assess spatial memory.	mRNA of ZO-1, VE-cadherin, Occludin, LRP-1, Mfsd2a, RAGE, AQP4 and Aβ42	Chronic alcohol intake altered spatial memory and exacerbated cognitive decline in APPswe/PS1dE9 mice. Alcohol decreased mRNA levels of functional and structural proteins in APPswe/PS1dE9 mice brains, with no changes in Aβ42 levels. **Conclusion:** Long-term intake of alcohol aggravates cognitive decline by causing neural lesions and dysregulating the structure and function of the BBB.	[32]
3xTg mice Age (start): P25–55 Age (end): ~ 6–12 months	25% alcohol (5 g/kg/d) orally in 2-day on/2-day off AIE regimen from P25 to P55. Unperturbed from P55–P200, followed by behavioral testing in adulthood (P200–270) and sacrifice	Open field test to assess locomotion. Novel object recognition test to assess recognition memory. Three-chamber social choice test. Acoustic startle test (pre-pulse inhibition). Morris water maze test to assess spatial memory. Sleep pattern assessment using Piezo sleep system	Aβ42, p-tau Thr181, IL-1β, MCP-1, IL-6, IFNα, TNFα, TLR4, and microglial DAM genes	AIE altered the mouse behavior and increased Aβ42, p-tau (Thr181), and various cytokines in female 3xTg mice brains. AIE also altered the expression of microglial DAM genes in female 3xTg mice. **Conclusion:** Alcohol intake in adolescence promotes the early onset of AD pathology by triggering proinflammatory microglial signaling.	[33]
APPswe/PS1dE9 mice Age (start): 6/12 months Age (end): 7/13 months	Alcohol (2.5 g/kg, intraperitoneally) intermittently over 6 weeks	Hebb–William’s maze to evaluate spatial memory.	Aβ42 and total hippocampal RNA and protein levels of CB2, DAGLα, and MAGL	Alcohol binge worsened cognitive impairment, accelerated AD pathology, and increased Aβ42 levels. Alcohol lowered CB2 RNA levels and increased the expressions of MAGL, with no change in DAGLα. **Conclusion:** Binge alcohol drinking during adolescence alters hippocampal ECS activity in adult mice. These altered ECS conditions could contribute to memory impairment. Binge alcohol also accelerates hippocampal Aβ in AD mice.	[34]
APP/PS1 mice Age (start): ~2 months Age (end): ~3 months	0.5, 1, 2, 3, and 4% *w*/*v* alcohol-containing liquid diet daily for 5 weeks	Y-maze test to evaluate spatial memory.	Aβ40, Aβ42, β-APP α, γ, and β-secretase activity	High dose (2–4%) of alcohol altered spatial memory, increased Aβ42, Aβ40, and β-APP by altering the γ- and β-secretase activities in the APP/PS1 mice. Low dose (0.5–1%) of alcohol had a protective effect in APP/PS1 mice. **Conclusion:** Dose-dependent effects of chronic alcohol intake such that higher doses of alcohol (2–4%) aggravate Aβ pathology and lower doses of alcohol (0.5–1%) reduce Aβ pathology.	[35]
3xTg mice Age (start): ~4 months Age (end): ~6 months	6% alcohol in drinking water 3 times per week for 2 months	Barnes maze test to assess spatial memory.		3xTg mice displayed improved working memory and altered Aβ aggregation with alcohol consumption. **Conclusion:** Alcohol consumption protects against Aβ-induced toxicity.	[36]
Tg2576 mice Age (start): ~4 months Age (end): ~11 months	Alcohol (6% in drinking water) over 7 months in the form of Cabernet Sauvignon (red wine) or alcohol	Barnes maze test to assess spatial memory.	Aβ40 and Aβ42, and liver panel enzymes (AST, ALT) to assess liver damage	Cabernet Sauvignon improved spatial memory and reduced neocortical Aβ40 and Aβ42 or AD-type plaque burden in the Tg2576 mice. Alcohol treatment had no effect on any of the measured parameters. Cabernet Sauvignon or alcohol did not change the serum levels of bilirubin, AST, and ALT in the Tg2576 mice. **Conclusion:** Cabernet Sauvignon at moderate doses protects from Aβ-induced neurotoxicity and improves cognitive recognition in the Tg2576 mice, which may be due to the antioxidants and polyphenols such as resveratrol.	[37]

Aβ, Amyloid beta; AD, Alzheimer’s disease; AIE, adolescence intermittent ethanol; ALT, alanine aminotransferase; APP, amyloid precursor protein; APP-CTF, C-terminal fragments of the amyloid precursor protein; AQP4, aquaporin-4; AST, aspartate aminotransferase; BACE-1, β-Site APP-cleaving enzyme 1; CB2, cannabinoid receptor; DAGLα, diacylglycerol lipases; DAM, disease-associated microglia; ECS, endocannabinoid system; ENT, entorhinal cortex; ERK, extracellular signal-regulated kinase ½; GABAA, γ-aminobutyric acid type-A; GSK3α, glycogen synthase kinases α; IGF1R, insulin-like growth factor1 receptor; IFNα, interferon alpha; IL-1β, interleukin-1β; IL-6, interleukin 6; IR, insulin receptor; IRS1, insulin receptor substrate-1; LEC, lateral entorhinal cortex; LRP-1, low density lipoprotein receptor-related protein-1; MAGL, monoacylglycerol lipase; Mfsd2a, major facilitator superfamily domain containing protein-2a; MAPK, mitogen activated protein kinase; MCP-1, monocyte chemoattractant protein-1; mPFC, medial prefrontal cortex; mTOR, mammalian target of rapamycin; NMDA, N-methyl-D-aspartate receptor; PS1, presenilin 1; PTEN, phosphatase and tensin homolog; RAGE, receptor for advanced glycation end products; TLR4, Toll-like receptor 4; Tg, transgenic; TNFα, tumor necrosis factor alpha; WT, wildtype; ZO-1, zonula occludens-1.

#### 3.2.2. Chronic Alcohol Feeding to Young APP23/PS45 AD Mice

Chronic alcohol treatment (20% *v*/*v* in water + 0.07% saccharin *w*/*v* using the drinking in the dark regimen) of six-week-old 2xTg-APP23/PS45 mice that carry mutations in human APP and PSEN1 (sex not mentioned) for four weeks increased APP processing and β-site APP cleaving enzyme 1 (BACE-1) levels, which enhanced Aβ production and aggravated cognitive dysfunction [30].

#### 3.2.3. Chronic Alcohol Feeding to Mature-Adult APP/PS1 AD Mice

In a study with 5.5-month-old male APP/PS1 AD mice mutated with human APP and PSEN1 genes, chronic alcohol feeding (20% *w*/*v* in water for 12 h/day for four consecutive days during the dark cycle) via a two-bottle choice drinking paradigm for 10 weeks resulted in brain atrophy and increased the frequency of smaller Aβ plaques in the APP/PS1 AD mice [31]. Chronic alcohol exposure altered glucose homeostasis, behavioral activity, hippocampal Aβ40 levels, mRNA levels of N-methyl-D-aspartate receptor (NMDA), and γ-aminobutyric acid type-A (GABA_A_) receptor in the APP/PS1 AD mice. Overall, alcohol altered cerebral metabolism and caused a shift of the brain’s excitatory/inhibitory balance that could potentially trigger Aβ deposition and aggravate AD pathology.

#### 3.2.4. Chronic Binge Alcohol Administration to Mature-Adult APP/PS1 AD Mice

Oral administration of alcohol once daily (likely by gavage but not clearly stated in the paper) for 60 days at a dose of 10 mL/kg to four-month-old male 2xTg-APPswe/PS1dE9 mice expressing chimeric mouse/human APP and human exon-9-deleted PSEN1 mutations aggravated cognitive decline by dysregulating the functional and structural integrity of the blood–brain barrier (BBB) [32].

#### 3.2.5. Alcohol Feeding to Adolescent 3xTg-AD Mice

Adolescent intermittent ethanol (AIE) treatment regimen (2 days on/2 days off) at a dose of 5 g/kg/d (25% *w*/*v* in water) by oral gavage in male and female 3xTg-AD mice from postnatal 25th to 55th day, followed by a period of being unperturbed for the next 145 days, showed an increased cognitive decline, significant increase in Aβ and tau pathology, and proinflammatory cytokines in adult female 3xTg-AD mice. Notably, this alcohol dosing regimen is expected to result in blood alcohol levels similar to binge drinking in human adolescents [33]. There was no significant change in the behavioral activity, Aβ and tau pathology, and proinflammatory cytokines in the male 3xTg-AD mice that underwent the AIE treatment, suggesting that adolescent binge alcohol enhances the risk of AD pathology in females in adulthood [33].

#### 3.2.6. Chronic Binge Alcohol Administration to Adolescent APP/PS1 AD Mice

Studies in male and female APPswe/PS1dE9 2xTg AD mice showed that chronic binge alcohol administration to adolescent mice (2.5 g/kg, i.p., four days a week over the adolescent period), experienced accelerated AD pathology during adulthood [34]. Chronic binge alcohol consumption increased Aβ42 levels in the hippocampus at 6 and 12 months of age and induced long-term cognitive impairment to a greater extent when administered to 6-month-old mice than when administered to 12-month-old mice [34].

Taken together, chronic alcohol feeding using various models that mimic liver injury observed with chronic moderate alcohol intake in humans appears to largely promote AD pathology in AD mice.

### 3.3. Studies Showing a Dose-Dependent Effect of Alcohol on AD Pathology

As previously mentioned, epidemiological studies have suggested that low alcohol intake may protect against AD, while heavy alcohol intake may promote it. One study examining alcohol dosing in APP/PS1 AD mice seems to support these contrasting effects of low and high alcohol doses on AD pathology [35]. In this study, the dose-dependent effects of alcohol on AD pathology were examined in two-month-old APP/PS1 2xTg AD male mice treated with a liquid diet containing different concentrations of alcohol (0.5, 1, 2, 3, and 4% *w*/*v* equivalent to 2.2, 4.5, 9, 13, and 18 g/kg/d) daily for five weeks. A significant impairment in learning and memory was observed at higher alcohol doses (2–4% *w*/*v* alcohol in liquid diet); however, lower doses (0.5–1% *w*/*v* alcohol in liquid diet) of alcohol improved memory function. In addition, higher alcohol doses (2–4% *w*/*v* alcohol in liquid diet) increased Aβ42, Aβ40, and β-APP levels, whereas there was a significant reduction in these parameters with lower doses (0.5–1% *w*/*v* alcohol in liquid diet) of alcohol. Finally, higher doses (2–4% *w*/*v*) of alcohol increased γ- and β-secretase activity, while lower doses (0.5–1% *w*/*v*) of alcohol significantly lowered γ- and β-secretase activity.

Two studies have explored the effect of low doses of alcohol on AD pathology. Four-month-old 3xTg-AD mice (sex not mentioned) that were fed alcohol (6% in drinking water—much lower than 20–25% alcohol in drinking water in most studies described above) three times per week for two months exhibited improvement in working memory [36]. This study did not examine Aβ or tau pathology and focused on the neuroprotective effects of low-dose alcohol, which will be discussed in the next section. Another study fed alcohol or Cabernet Sauvignon in drinking water (alcohol content ~6% in water equivalent to 8 g/kg/d) to four-month-old female mutant human APP expressing Tg2576 mice for seven months [37]. Alcohol feeding did not have any effect on cognitive function or Aβ in the AD mice; however, significant protective effects of Cabernet Sauvignon on working memory and Aβ pathology were observed. The protective effects of Cabernet Sauvignon observed in the study may be attributed to extracts in red wine (antioxidants and polyphenols such as resveratrol). Notably, no changes in liver function were reported in this study.

Collectively, results from alcohol-feeding studies in AD mice show the varying impact of chronic alcohol consumption on AD pathology that may be attributed to different alcohol concentrations and resultant liver injury, duration of alcohol consumption, sex, age at initiation of treatment, and/or the rodent model used (Table 1). Notably, the AD mice used have different genetic backgrounds, which may significantly impact alcohol consumption [38]. Overall, alcohol feeding to AD mice at doses known to cause significant liver steatosis seems to accelerate AD pathology, although information pertaining to liver damage was largely not reported in the reviewed studies. A few studies seem to show some protective or no effects of low-dose alcohol on AD pathology. However, since these studies remain low in number, further experiments are needed to support the protective effects of low-dose alcohol on AD pathology in rodent models.

## 4. Alcohol Intake and AD: Evidence from Cell Culture Studies

A number of cell culture studies have demonstrated a potential link between alcohol and AD and have helped to provide mechanistic insights into the impact of alcohol on AD pathology. It should, however, be noted that cell culture studies involving alcohol doses are relatively difficult to extrapolate to in vivo doses due to alcohol’s rapid evaporation in cell culture conditions (37 °C).

Using stable human cells lines (HEK293 embryonic or SH-SY5Y neuroblastoma cells) that express mutant human APP and BACE-1, studies show that alcohol at different concentrations (up to 139 mM) increased the expression of APP, BACE-1, Aβ42, and Aβ40, demonstrating the ability of alcohol to increase APP processing and Aβ production in vitro [30] (Table 2). Similarly, in a study using human neuroblastoma cells (SK-N-MC), exposure to alcohol (34, 69, and 103 mM; notably, blood alcohol concentrations in alcohol abusers reach or exceed 69 mM) and acetaldehyde (54, 107, and 215 µM) showed a dose-dependent increase in the expression of BACE-1 and the C99 fragment of APP. The latter is cleaved by γ-secretase to produce Aβ. The increased expression of BACE-1 and C99 fragments was attenuated in cells pre-treated with sodium azide, a catalase inhibitor to inhibit ethanol metabolism, suggesting the role of catalase as the major metabolic enzyme involved in the increased expression of BACE-1 by alcohol [39]. Furthermore, treatment of N2a-APP cells (mouse neuroblastoma N2a cells that express mutant human APP) with alcohol (412 mM) and APOE4 (7.5 µg/mL) exacerbated neuronal apoptosis and elevated cellular oxidative stress. The neurotoxic effects of APOE4 and alcohol were found to be synergistic, suggesting an AD-potentiating role of alcohol in APOE4 carriers [40].

Besides increasing APP processing and Aβ synthesis, alcohol intake can also impact Aβ clearance. In a study using primary rat microglial cells, acute alcohol treatment (75 mM) for 24 h reduced the phagocytosis of fluorescently labeled oligomeric Aβ42, which may increase Aβ load by impairing microglia mediated Aβ clearance [41]. Notably, this study utilized modular chambers to prevent alcohol evaporation. With respect to the effect of alcohol on tau pathology, treatment of human neuroblastoma cells that stably express the 4R0N isoform of human tau with alcohol (27–109 mM) showed a dose-dependent accumulation of tau and reduction in cell viability, which was attributed to a reduced tau clearance [42].

In contrast, some studies suggest that lower doses of alcohol and/or alcohol pre-conditioning may protect against Aβ-induced toxicity in cell culture models. Pre-incubating Aβ with 10 mM alcohol prevented the formation of toxic Aβ dimers and reduced Aβ-induced toxicity in HEK and PC-12 cells [43]. Similarly, the co-incubation of Aβ (1–5 µM) with alcohol (10 mM) reduced Aβ-induced synapto-toxicity and inhibited Aβ aggregation in primary rat hippocampal neurons [36] (Table 2). Accordingly, the pre-treatment of mouse primary cortical neurons with 10 mM alcohol and hippocampal–entorhinal slice cultures with alcohol (20–30 mM) reduced Aβ-induced neurotoxicity [44,45].

**Table 2:** List of cell culture studies reporting the effects of alcohol on AD pathology.

Invitro Model	Assay	Biomarkers Measured	Outcome	Ref. #
2EB2 cells (HEK293 cells overexpressing human Swedish APP and BACE-1), 20E2 cells (expressing human Swedish APP in HEK293), SH105 cells, and SH-SY5Y cells (human neuroblastoma cell line expressing BACE-1)	Cells were treated with 0-, 9-, 17-, 35-, 69-, and 139 mM alcohol for 48 h.	Protein levels of APP, APP-CTFs, ADAM10, BACE-1, and PS1 in 2EB2 cell lysates. Aβ40 and Aβ42 in the media of 20E2 cells. APP and BACE-1 in SH105 and SH-SY5Y cells.	Alcohol treatment increased the expression of: APP, APP-CTFs, and BACE-1 in 2EB2 cells, Aβ40 and Aβ42 levels in 20E2 cell lysates, and APP and BACE-1 in SH105 and SH-SY5Y neuronal cell lines. **Conclusion:** Alcohol exposure promotes APP processing and aggravates AD-associated phenotypes in a dose-dependent manner.	[30]
Primary rat hippocampal 10–12-day cultures	Cells incubated with Aβ (1 and 5 µM) and alcohol (1–100 mM) at 37 °C for 30 min.	Aβ, α-tubulin, synaptophysin, and synaptic vesicle 2 were detected by Western blotting.	Low concentrations (10mM) of alcohol reduced Aβ oligomer-induced neurotoxicity. Alcohol decreased the association of Aβ oligomers with neurons and increased Aβ disaggregation. **Conclusion:** These results may explain the lower risk of AD with low alcohol consumption.	[36]
SK-N-MC cells (human neuroblastoma cells)	Cells incubated with serum-free medium, later exposed to alcohol (0–103 mM) and acetaldehyde (0–215 µM) for 24 h. Parafilm-sealed plates were used to minimize alcohol evaporation.	Aβ and PGE2 levels in the cell culture media after alcohol exposure were measured. BACE-1, C-99, and intracellular ROS levels were also measured.	Alcohol increased BACE-1, C-99 fragment of APP, Aβ secretion, ROS, and PGE2 levels. Acetaldehyde increased BACE-1 expression. **Conclusion:** Alcohol-induced ER stress affects the PKA/CREB pathway via COX-2-mediated PGE2 formation, which leads to increased BACE-1 expression and Aβ production.	[39]
Mouse neuroblastoma N2a cell line expressing human APP (N2a-APP-695)	N2a-APP cells were incubated (16 h) with or without alcohol (130 mM/522 mM) in the presence of vehicle, APOE3 or APOE4 protein. Seal-plate film was used to minimize alcohol evaporation.	Cell viability, apoptotic cells, and cellular oxidative stress.	Synergistic neurotoxic effects of APOE4 and alcohol on N2a-APP cells. APOE4 exacerbated alcohol-induced neuronal apoptosis and cellular oxidative stress in N2a-APP cells. **Conclusion:** The study revealed the differential roles of APOE isoforms on alcohol-induced neurotoxicity, with APOE4 augmenting cellular oxidative stress and apoptotic cell death.	[40]
Primary rat microglial cells (prepared using frontal cortex of postnatal day 2 SD rats of both sexes)	Cells were incubated at 37 °C for 24 h with 75 mM alcohol along with a mixture of proinflammatory cytokines (TNFα, 10 ng/mL, IL-1β, 10 ng/mL, and IFNγ, 10 IU/mL) in a chamber flushed with 5% CO_2_, 21% O_2_, and balanced N_2_. Modular chambers were used to minimize alcohol evaporation.	Aβ phagocytosis was assessed. Nitrite production measured (to assess inflammatory activation of microglia). Microglial mRNA levels were measured.	Exposure of microglial cells to alcohol reduced Aβ42 phagocytosis and increased nitrite levels. Alcohol increased changes in phagocytosis-related mRNAs. **Conclusion:** Alcohol significantly suppresses Aβ42 phagocytosis in rat microglial cells.	[41]
M1C cells (human neuroblastoma BE2-M17D cell line expressing the 4R0N isoform of human tau)	Cells were incubated with different concentrations of alcohol (27, 54, and 109 mM) for up to 4 days.	Tau mRNA and protein levels were measured. Cell viability was assessed. Calpain, cathepsin B, and cathepsin D activity were measured.	Alcohol increased tau protein, decreased cell viability, and reduced calpain activity but had no effect on the activity of cathepsin B and D. **Conclusion:** Alcohol causes dose-dependent tau accumulation and reduced cell viability in the M1C cells.	[42]
HEK293 cells (human embryonic kidney cells), PC12 cells (pheochromocytoma of the rat adrenal medulla cells)	Cells were grown in 48-well plates and exposed for 24 h to Aβ (0.05 to 10 µM) in cell culture medium containing alcohol (0.1 mM, 10 mM, and 50 mM).	Aβ aggregation and cell viability were assessed.	Alcohol reduced Aβ aggregation and the toxic effects of Aβ. **Conclusion**: Alcohol protects against Aβ toxicity by destabilizing the salt bridge formed between Asp 23 and Lys 28 of Aβ that prevents the formation of complex Aβ fibers.	[43]
Rat hippocampal-entorhinal brain slice cultures	3-week hippocampal-entorhinal slices cultured in 6-well plates were exposed to 30 mM alcohol, incubated for 6 days in a desiccator with 90 mM alcohol to minimize alcohol loss. Later the cultures were treated with pre-aggregated Aβ42 (20 µM) for 24 h.	Cell lysis and cell death were assessed by determining lactate dehydrogenase (LDH) activity. Neuronal cell death in the brain slices was visualized using propidium iodide (PI) along with Aβ42 over 24 h incubation period. Apoptotic DNA fragmentation was detected with bisbenzimide (DNA-binding dye Hoechst 33,342) for 2 min. Inducible heat shock protein70 (hsp70) in the brain slice mixture was determined by ELISA.	Moderate alcohol exposure in the hippocampal brain slice cultures protected the neuronal cells against Aβ42 neurotoxicity by lowering LDH activity and by increasing hsp70 levels. **Conclusion:** Moderate alcohol exposure protects the hippocampal-entorhinal cortical neurons against Aβ toxicity.	[44]
Primary neuronal cultures (cortical and hippocampal neurons obtained from mouse brains)	Neuronal cultures pre-treated with different concentrations of alcohol (4–20 mM) for 1 h were exposed to synthetic peptide biotin-Aβ42 for 1 to 24 h.	Biotin-Aβ42, synaptophysin, and activated cytoplasmic phospholipase-A2 (cPLA2) levels were measured. Free calcium levels in the synaptosomes were also measured.	Alcohol (4–20 mM) protected the neurons against Aβ-induced synapse damage, reduced activated cPLA2, and increased the Aβ-induced increase in calcium levels in the synaptosomes. **Conclusion:** Low alcohol concentrations (9 mM) protect the cortical and hippocampal neurons against Aβ42 toxicity by inhibiting cPLA2 activation at the synapse.	[45]

Aβ, Amyloid beta; AD, Alzheimer’s disease; ADAM10, A disintegrin and metalloproteinase domain-containing protein 10; ApoE4, ε4 allele of apolipoprotein E; APP, amyloid precursor protein; APP-CTFs, C-terminal fragments of APP; BACE-1, β-Site APP-cleaving enzyme 1; COX-2, cyclooxygenase-2; CO_2_, carbon dioxide; cPLA2, cytoplasmic phospholipase-A2; CREB, cyclic adenosine monophosphate (cAMP) response element-binding protein; ELISA, enzyme-linked immunosorbent assay; ER, endoplasmic reticulum; hsp70, heat shock protein70; IL-1β, interleukin-1β; IFNγ, interferon-gamma; LDH, lactate dehydrogenase; N_2_, nitrogen; O_2_, oxygen; PGE2, prostaglandin E2; PI, propidium iodide; PKA, protein kinase A; PS1, presenilin 1; ROS, reactive oxygen species; TNFα, tumor necrosis factor alpha.

## 5. Alcohol Use and AD: Mechanistic Insights into the Brain

**Figure 1:**
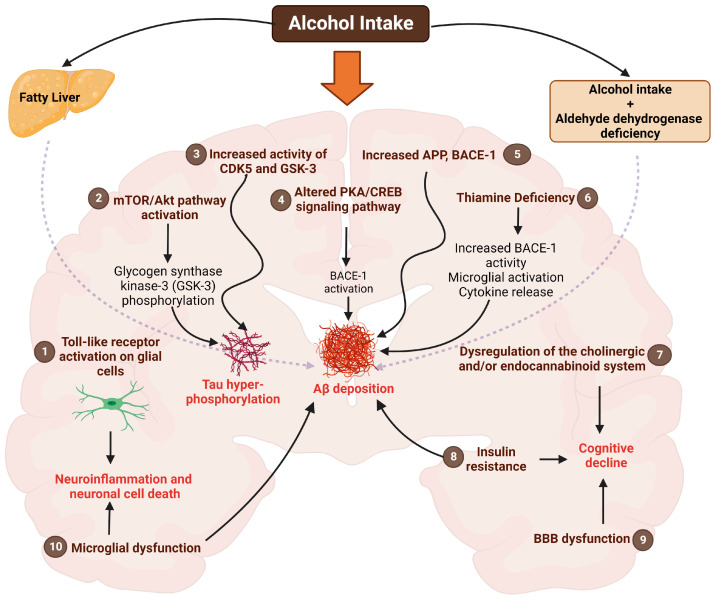
Schematic representation of the potential mechanisms by which alcohol modulates Alzheimer’s disease (AD) pathology. Alcohol consumption can activate Toll-like receptors, which trigger the innate immune response, aggravate microglial activation, and cause neuroinflammation and neuronal cell death (1). Chronic alcohol intake can also activate the mTOR/Akt pathway, which phosphorylates GSK-3 and causes tau hyperphosphorylation, leading to neurodegeneration (2). Alcohol is also known to upregulate the CDK5 and GSK-3β kinase activities, leading to tau hyperphosphorylation (3). Alcohol is additionally known to alter the PKA/CREB signaling pathway and increase BACE-1 activity, leading to the upregulation and deposition of Aβ (4). Similarly, chronic alcohol intake can increase APP levels and BACE-1 activity, leading to increased Aβ production (5). Additionally, alcohol intake causes thiamine deficiency, which activates BACE-1 and microglial cells and triggers proinflammatory cytokine release, leading to Aβ accumulation (6). Chronic alcohol intake also dysregulates the cholinergic and endocannabinoid system, leading to cognitive decline (7). Chronic alcohol intake is additionally known to disrupt insulin signaling and impair the cholinergic system, increasing Aβ deposition, neuronal loss, and cognitive deficits (8). Alcohol intake is known to cause BBB (9) and microglial (10) dysfunction, which are significant drivers of AD pathology and cognitive deficits. Apart from these cerebral effects of alcohol, other potential mechanisms by which alcohol can modulate AD pathology include an increase in Aβ accumulation with aldehyde dehydrogenase deficiency and alterations in hepatic Aβ processing associated with liver injury. The latter may act as a source of brain Aβ. The figure was created using BioRender.com. Aβ, Amyloid beta; AD, Alzheimer’s disease; APP, amyloid precursor protein; BACE-1, β-Site APP-cleaving enzyme 1; BBB, blood–brain barrier; CDK5, cyclin-dependent kinase-5; CREB, cyclic adenosine monophosphate (cAMP) response element-binding protein; GSK3, glycogen synthase kinases; mTOR, mammalian target of rapamycin; PKA, protein kinase A.

Alcohol can cross the BBB and is known to be a direct neurotoxin that can cause abnormalities in brain structure (i.e., leads to brain shrinkage/atrophy, demyelination) and alters brain function (i.e., causes intellectual and memory impairment) following chronic consumption [46]. For example, an analysis of the brains of alcoholics showed atrophy or a loss of volume in various areas of the brain, including the cortex and hippocampus [47,48,49]. The different pathways and targets of alcohol-mediated neurotoxicity in AD that have been postulated are summarized in Figure 1 and include Toll-like receptors, Akt/mTOR pathway, cAMP response element-binding protein (CREB) phosphorylation pathway, glycogen synthase kinase 3-β (GSK3β), cyclin-dependent kinase-5 (CDK5), and insulin-like growth factor type-1 (IGF) receptor genes highly expressed in the various regions of the brain, such as the hypothalamus, temporal lobe, and cerebellum [50,51,52,53].

### 5.1. Toll-like Receptors (TLRs)

TLRs are pattern recognition receptors that initiate innate immune signaling, releasing cytokines and chemokines in response to an abnormal stimulus, including pathogens or injury. In the central nervous system (CNS), TLRs are mainly expressed by the glial cells, including microglia, the resident macrophages in the brain. It has been shown that alcohol at 10, 50, and 100 mM can activate TLR4 in cultured glial cells, suggesting that TLR4 plays a role in alcohol-induced inflammation. Furthermore, glial cell activation in response to alcohol treatment releases proinflammatory mediators and neuronal cell death [54,55]. In AD, TLR2 and TLR4 are activated in response to Aβ to trigger microglial phagocytosis, oxidative stress, and neuroinflammation [51,56]. Interestingly, a TLR4 polymorphism (Asp299Gly) is associated with a reduced risk of late-onset AD [57]. Additionally, a study on a postmortem human alcoholic brain showed an increase in the mRNA levels of TLR7 and increased microglial activation. TLR7 signaling triggers the neuroimmune response by activating nuclear factor-κB (NFκB) or interferon regulatory factor-7 (IRF-7) and causes neurodegeneration [58]. Taken together, TLRs are activated following alcohol intake and in the AD brain [51], and it is postulated that TLR activation by alcohol may aggravate microglial activation, neuroinflammation, and neuronal cell death in AD [53].

### 5.2. Akt/mTOR Pathway

The Akt/mTOR pathway is an important cell signaling pathway regulating various cellular functions, including glucose uptake and metabolism in the brain to modulate cell growth, proliferation, and autophagy [29]. The core components, Akt (serine/threonine kinase) and mTOR (mammalian target of rapamycin) are known to be activated in the nucleus of accumbens (Nac) with chronic alcohol consumption in rodents [59]. Activation of Akt phosphorylates glycogen synthase kinase-3 (GSK-3), an alcohol-sensitive protein, which in turn leads to tau hyperphosphorylation and neurodegeneration [29,53]. It is known that acute and chronic alcohol consumption dysregulates the Akt/mTOR signaling pathway by altering various phosphoproteins, including insulin receptor (IR), insulin receptor substrate 1 (IRS1), insulin-like growth factor 1 (IGF-1) receptor, phosphatase and tensin homolog (PTEN), extracellular signal-regulated kinases (ERK), mTOR, 70-kDa ribosomal protein S6 kinase (p70S6K), and ribosomal protein S6 (RPS6) in different regions of the brain, including the hippocampus and cortex, that lead to neurodegeneration [29].

### 5.3. Cyclic-AMP Response Element Binding Protein (CREB) Pathway

CREB is a widely expressed transcriptional factor in the CNS that plays a vital role in regulating neuronal growth, survival, neurogenesis, synaptic function, and memory formation in the brain. Phosphorylation of CREB at serine-133 (S133) is essential for the regulation of its transcriptional activity, and the expression of the CREB regulating gene is downregulated in AD [60,61]. Phosphorylated CREB is known to downregulate the pathological biomarkers of AD, including Aβ deposition and tau aggregation, and upregulates the expression of brain-derived neurotrophic factor (BDNF), the key neurotrophic factor that plays a vital role in neuronal survival and growth. The downregulation of BDNF expression leads to cognitive impairment and promotes neuronal dysfunction in AD [61]. It is postulated that alcohol induces ROS production, leading to the phosphorylation of eIF2α, which stimulates the expression of COX-2. The induced COX-2 stimulates PGE2 synthesis that binds with the EP-2 receptor, leading to the downregulation of PKA that impairs the phosphorylation of CREB. The altered PKA/CREB signaling triggers BACE-1 expression and upregulates Aβ production, leading to the progression of AD pathology (Figure 1) [39]. Further, the treatment of human neuroblastoma cells (SH-SY5Y) and human fetal brain neural stem cell (hNSC)-derived primary neurons with varying concentrations of alcohol (0–500 mM) for 24 h decreases the phosphorylation of CREB in a dose-dependent manner at doses higher than 300 mM, leading to mitochondrial dysfunction and neurodegeneration [62].

### 5.4. CDK5 and GSK Pathway

GSK3β and CDK5, a member of the cyclin-dependent kinases (CDKs) and also known as tau protein kinase II, are kinases implicated in the hyperphosphorylation of the microtubule-associated protein tau [63]. The activity of CDK5 is regulated by its activators, including p35, under physiological conditions. However, under pathological conditions, p35 is cleaved to its N-terminal truncated form, p25, which over-activates CDK5, and this cleavage is mediated by calcium-activated neutral proteases, the calpains [64]. CDK5 activity has been shown to be increased in the AD brain, and it has been suggested that high levels of p25 promote tau hyperphosphorylation and neurodegeneration in AD [63,64]. Chronic alcohol intake has been shown to increase CDK5 and calpain activity [65]. Further, alcohol (2.5 g/kg subcutaneously at 0 and 2 h) in postnatal (P7) mice enhances the conversion of p35 to p25 and causes hyperphosphorylation of tau [66,67]. Similarly, alcohol feeding to rats (4 mL/kg for 30 days) increases GSK3β activity and tau hyperphosphorylation at Ser 199, Ser 396, and Ser 404 [68,69]. Collectively, the increased activity of CDK5 and GSK3β with alcohol intake may contribute to abnormal tau phosphorylation and NFT formation in AD.

### 5.5. Insulin Resistance

Insulin is a polypeptide hormone primarily secreted by the pancreatic β-cells and regulates blood glucose concentrations in the peripheral organs [70]. Increasing evidence of the localization of insulin, its receptors, and function in the brain tissue has highlighted the central effects of insulin. Insulin and insulin-like growth factor-type-I (IGF-1) receptors are abundantly expressed in the CNS and mediate important functions, including neuronal cell growth, survival, plasticity, and metabolic functions [71]. Alcohol is known to suppress insulin and IGF-1 signaling by inhibiting the insulin receptor substrates (IRS-1), IGF-1 receptors, and phosphatidylinositol-3 (PI3) kinase signaling in neuronal cells [72]. Chronic alcohol (9.25% *v*/*v* for six weeks) intake-mediated neurodegeneration in adult rats is associated with a brain-region-specific reduction in the expression of insulin receptors, IGF-I and IGF-II receptors, choline acetyltransferase, and impaired insulin and IGF-1 binding in the CNS [72,73]. Similarly, post-mitotic rat cerebellar neuronal (rCBN) cultures exposed to alcohol (50 mM) for four days experienced an inhibition of insulin-stimulated mitochondrial function [74]. Further, chronic alcohol exposure in rats inhibited insulin and IGF-1 signaling in the brain, leading to impaired acetylcholine homeostasis and neuronal loss [75]. These studies collectively indicate that chronic alcohol intake can disrupt insulin signaling and result in cholinergic deficits. With respect to AD, insulin in the brain is known to promote the regulation of Aβ clearance, tau phosphorylation, cerebral blood flow, oxidative stress, lipid metabolism, and memory formation [76,77]. Therefore, any disruption in insulin signaling in the brain has the potential to contribute to AD pathology. Accordingly, AD brains are reportedly insulin resistant, and studies show disrupted insulin and IGF-1 signaling in AD brains [78,79]. Chronic alcohol intake-triggered insulin disruption may thus potentiate or instigate AD pathology.

### 5.6. Thiamine Deficiency

Thiamine (vitamin B1) is a water-soluble essential micronutrient that is a cofactor for major enzyme systems associated with the metabolism of glucose, amino acids, and lipids [80]. Alcohol abuse is associated with thiamine deficiency, which cascades into a series of events such as a mild-to-chronic impairment of oxidative metabolism, neuroinflammation, microglial activation, and the induction of oxidative stress and endoplasmic reticulum (ER) stress in the brain, leading to neuronal loss in specific regions of the brain [80,81]. Accordingly, thiamine deficiency has been shown to aggravate Aβ plaque pathology and promote tau phosphorylation in transgenic AD mice [82]. Thiamine deficiency is known to increase the expression of BACE-1, activate the glial fibrillary acidic protein, decrease the activity of the alpha-ketoglutarate dehydrogenase complex, and trigger the production of proinflammatory cytokines such as TNF-α in AD mice, leading to the accumulation of Aβ plaques [83].

### 5.7. Endocannabinoid System

The endocannabinoid signaling system (ECS) comprises three key elements: the cannabinoid receptors (CB1 and CB2), endocannabinoid molecules/ligands (N-arachidonic-ethanolamine (AEA), 2-arachidonilglycerol (2-AG)) and their corresponding ligand synthesizing and degrading enzymes (N-acyl-phosphatidylethanolamine (NAPE) and fatty acid amide hydrolase (FAAH) for AEA and 1,2-diacylglycerol lipase-α (DAGLα) and monoacylglycerol lipase (MAGL) for 2-AG) [84].

The ECS plays a vital role in the brain, regulating neuronal communication, neuroinflammation, and behavior [85]. Alcohol is known to alter the ECS, and intermittent alcohol administration to rats (3 g/kg, i.p.) for four weeks alters the expression of endocannabinoid receptors and enzymes, impacting cognition and behavior [85]. Studies report that acute alcohol exposure is known to increase endocannabinoid levels (2-AG) in the brain [86], whereas chronic alcohol consumption results in the depletion of endocannabinoid levels (AEA and 2-AG) in the midbrain [87] that may adversely affect the different physiological processes regulated by the ECS. Studies report that Aβ-induced hippocampal neurodegeneration is associated with increased 2AG production, which is considered to be a potential defense mechanism to counter neurodegeneration in the AD brain [88]. Taken together, it can be hypothesized that chronic alcohol intake reduces endocannabinoid (2AG) levels, which may aggravate AD pathology [34]. Analysis of AD patients’ brains has revealed that CB2 receptors were selectively overexpressed in cells associated with Aβ-enriched neuritic plaques. The intracerebroventricular administration of Aβ to rats has been shown to augment hippocampal CB2 receptor, MAGL, 2-AG, and DAGLα levels. Additionally, CB2 receptor stimulation in rat hippocampal neuronal cultures (via CB2 receptor agonist 2-AG or the selective inhibition of the 2-AG degrading enzyme, MAGL) inhibits Aβ-induced neurodegeneration [89]. Likewise, CB2 receptor activation is known to protect against Aβ-induced neuronal toxicity in rats [90]. Comparable effects have been noted in rodent models of AD, where the stimulation of the CB2 receptor or inhibition of MAGL resulted in Aβ reduction in the hippocampus and showed a significant improvement in cognitive function [89,91,92,93,94,95].

### 5.8. Cholinergic System

Acetylcholine plays a vital role in learning and memory, and deficits in the cholinergic system in AD have been well documented. Chronic alcohol abuse leads to the loss of cholinergic neurons and inhibits acetylcholine release, leading to cognitive impairment [96,97]. Further, the chronic alcohol (5–20% *v*/*v* for six months) feeding of rats results in a reduction in hippocampal cholinergic innervations characterized by the loss of cholinergic fibers and loss of hippocampal neurons, which in turn may lead to cognitive dysfunction [98]. Accordingly, the chronic administration of alcohol (5–10% *v*/*v* for up to 9 months) to rats showed a pronounced reduction in the activity of choline acetyltransferase, the enzyme that synthesizes acetylcholine, and a reduction in high-affinity choline uptake, the rate-limiting step in acetylcholine synthesis, in the frontal cortex and basal nucleus complex of the brain, leading to cholinergic degeneration and probable cognitive dysfunction [99]. Similarly, chronic alcohol consumption in humans decreased cholinergic neurons, depleted the activity of choline acetyltransferase in the frontal cortex, and led to cognitive impairment [99,100]. Overall, chronic alcohol consumption alters the cholinergic innervations in the brain. These deficits in the cholinergic system are postulated to be a unifying mechanism underlying alcohol use and AD [6,98].

### 5.9. Blood–Brain Barrier (BBB) Alterations

The BBB plays an essential role in the proper maintenance of the CNS by protecting the brain from the infiltration of pathogens, neurotoxic chemicals, and numerous macromolecules [101]. An impairment of BBB function and integrity in AD has been shown in multiple studies [102]. In fact, BBB dysfunction has been reported to be an early biomarker of cognitive decline in AD, independent of Aβ and tau measurements [103,104]. BBB dysfunction and breakdown can contribute to AD pathology and cognitive decline via different mechanisms, including a reduction in brain perfusion, alteration in the function and expression of BBB transporters and receptors, an infiltration of peripheral blood-derived products and neurotoxins, neuroinflammatory and neuroimmune responses, and eventual neuronal loss and cognitive decline [102]. For example, studies performed on rodent AD models show low levels of LRP1 and high levels of receptor for advanced glycation end products (RAGE) at the BBB; these alterations can increase Aβ load in the brain. Further, a loss of BBB integrity allows CNS leukocyte infiltration, capable of triggering a neuroimmune response and neurodegeneration [102]. With respect to alcohol, chronic intake has been suggested to weaken the BBB, and BBB dysfunction may thus be another important mechanism by which alcohol potentially promotes AD. In primary human brain microvascular endothelial cells (BMVEC), alcohol treatment has been shown to induce oxidative stress-caused loss of BBB integrity and enhance leukocyte migration across BMVEC monolayers [105]. Alcohol feeding to rats (Leiber DeCarli oral alcohol liquid diet with 5% *v*/*v* alcohol for nine weeks) resulted in the degradation of essential tight junction proteins, including occludin, claudin-5, and ZO-1 by matrix metalloproteinases (MMP)-3/9 [106]. Similarly, a chronic binge of alcohol (once daily for two months) to C57BL/6 and APPswe/PS1dE9 AD mice resulted in increased permeability of the BBB, likely due to downregulation of tight junction proteins (ZO-1, VE-cadherin, and occludin), and altered the levels of Aβ transporters LRP1 and RAGE [32]. Finally, a postmortem analysis of human alcoholic brains showed a reduced expression of structural proteins (claudin-5 and collagen-IV) and increased leukocyte infiltration (increased CD45 positive cells) in the dorsolateral prefrontal cortex. Further, BBB impairment seen with alcohol intake was mediated by TLR4 [107], the expression of which is upregulated by alcohol intake (see Section 5.1). Taken together, there is compelling evidence that alcohol intake can weaken the BBB, which has been demonstrated to be an important early event in AD development. Alcohol-induced BBB dysfunction may thus be an important pathway contributing to alcohol-dependent AD.

### 5.10. Microglia-Mediated Effects

Microglia, the primary immune cells of the CNS, respond to brain insult and play an essential role in immune surveillance, synaptic pruning, and the phagocytic removal of cellular debris, pathogens, and protein aggregates (including Aβ), neuromodulation, and overall brain homeostasis [108]. In the AD brain, microglia are found to be closely associated with Aβ plaques, and this association can trigger microglial activation and subsequent phagocytic removal of these protein aggregates [108]. Additionally, Aβ-plaque-associated microglia may limit plaque expansion and neuronal injury [109]. However, sustained microglial activation, as seen in the aging or AD brain, can cause an excessive release of cytokines and chemokines, which are known to contribute to the pathogenesis of AD [108]. In this regard, alcohol intake, either binge [110,111] or chronic [112,113], has been shown to result in some degree of microglial activation and functional deficits. Our recent work shows that intragastric feeding of alcohol to mice reduces microglial count in the brain [28]. This reduction in microglial number or function may disrupt CNS recovery and homeostasis. Accordingly, in experimental AD models, acute treatment of primary microglial cultures with high-dose alcohol (75 mM) reduced Aβ uptake and phagocytosis and may be the underlying mechanism of alcohol-mediated Aβ increase. Further, the treatment of 3xTg-AD mice with alcohol equivalent to adolescent binge alcohol dosing significantly altered the expression of disease-associated microglial genes, including Cst7, C3, and Tmem119, in female mice. These proinflammatory signatures appeared to drive AD pathology in the 3xTg-AD mice, highlighting the role of alcohol-induced microglial activation in AD pathogenesis [33].

### 5.11. Aldehyde Dehydrogenase (ALDH)

*ALDH* is one of the key enzymes involved in alcohol metabolism, and the ALDH2*2 mutation has been identified as a risk factor for AD [114]. ALDH is responsible for detoxifying acetaldehyde into acetate, and ALDH2*2 mutation is associated with a significant decrease in the capacity to metabolize acetaldehyde [115]. Chronic alcohol feeding (1 g/kg/d for 11 weeks) to mice deficient in ALDH activity increased brain Aβ42 compared with WT mice. This suggests that increased acetaldehyde levels due to insufficient ALDH activity following alcohol feeding may modulate pathways involved in Aβ processing [116]. Thus, acetaldehyde, a metabolite of alcohol metabolism and probable carcinogen (EPA classification) may play a role in promoting AD pathology caused by alcohol intake.

### 5.12. Aβ Production and Other Mechanisms

Besides the mechanisms mentioned above, chronic alcohol intake can modulate AD pathology by having a direct effect on Aβ production. Aβ synthesis occurs through the sequential cleavage of APP by BACE-1 and γ-secretase [117]. In male Sprague Dawley rats weighing around 150–170 g, a chronic alcohol administration (50 g alcohol/liter) for five weeks increased hippocampal APP and BACE-1 levels, proteins involved in Aβ production, compared to control-diet-fed rats, indicating that chronic alcohol consumption may increase amyloidosis [118]. Accordingly, in a recent study with APP/PS1 AD mice, the chronic intake of alcohol (3–4% in liquid diet) altered APP levels, increased BACE-1 activity, and increased Aβ levels in AD mice brains. In contrast, in the same study, chronic alcohol intake at lower doses (0.5–1%) reduced BACE-1 activity and suppressed Aβ production and deposition [35].

## 6. Alcohol Use and AD: Peripheral Effects of Alcohol

Peripheral organs such as the liver have a significant impact on brain health and, therefore, may impact the progression of AD pathology. Since alcohol intake most affects the liver, the primary site of alcohol metabolism [26,27], this section will focus on a possible liver-to-brain axis in AD that may be modulated by alcohol. As previously mentioned, alcohol intake causes alcoholic steatosis (fatty liver), alcoholic hepatitis (inflammation plus fatty liver), and cirrhosis (fibrosis due to significant hepatocyte death) [119,120]. Alcoholic cirrhosis is a risk factor for hepatocellular carcinoma (HCC), the cancer most associated with alcohol abuse. Peripheral organs such as the kidneys may undergo various stresses, including oxidative stress with alcohol intake, but do not develop extensive pathology such as the liver [121]. There may be multiple mechanisms by which the liver-to-brain axis may be modulated by alcohol, but our focus will be on Aβ homeostasis and inflammation.

### 6.1. Impact of Peripheral Aβ on AD

Peripheral Aβ is transported across the BBB by RAGE, and conversely, Aβ from the brain can be transported into the periphery by LRP1 [122]. Expression of RAGE and LRP1 in the brain endothelial cells that compose the BBB may help maintain Aβ homeostasis between the brain and periphery. While some studies have shown that modulating peripheral Aβ does not impact brain Aβ levels [123,124], other studies have shown a peripheral-to-central Aβ crosstalk [125] and that alterations in plasma Aβ levels may correlate with changes in brain Aβ pathology [126]. Similarly, peritoneal dialysis in patients was found to reduce Aβ pools in blood and brain interstitial fluid (ISF) [127]. Peritoneal dialysis of APP/PS1 mice (once a day for one month) was found to attenuate AD pathology, including tau hyperphosphorylation, glial activation, neuroinflammation, neuronal loss, and synaptic dysfunction [127]. Further, the whole blood exchange of AD mice (Tg2576) with blood from normal mice resulted in a significant reduction in the Aβ plaque load in the mice brains [128]. Taken together, peripheral Aβ pools appear to impact cerebral Aβ pools under certain conditions, and alterations in peripheral Aβ may impact the Aβ load in the brain.

### 6.2. Impact of the Liver on Peripheral Aβ Levels

Peripheral Aβ may originate from the brain through transport by LRP1 across the BBB, but many peripheral organs can produce Aβ, including the liver, pancreas, adipose tissue, and skeletal muscles [126]. It has recently been shown that glucose induces the release of Aβ from the pancreas along with insulin, which then triggers Aβ release from the liver, adipose tissue, and muscle [126]. Further, peripheral Aβ in circulation can be removed by the liver and, to a lesser extent, by the kidneys and spleen [129]. The liver contains receptors that can remove Aβ from the circulation, including LRP1, RAGE, and P-glycoprotein [130,131]. LRP1, however, appears to be the most important receptor for peripheral Aβ clearance by the liver, and its suppression can cause a dramatic decline in Aβ uptake by the liver [130]. Since the liver can both produce and remove Aβ from circulation, it is not entirely known whether it is a source or remover of peripheral Aβ under normal conditions. However, our recent study demonstrated that chronic alcohol feeding and the steatosis it produces likely make the liver a producer of Aβ in the periphery [28]. We observed that both the NIAAA alcohol model and intragastric alcohol feeding to WT mice (C57BL/6) for 4–6 weeks caused a decrease in LRP1 expression (~50%), increase in APP expression (~100%), and increased Aβ levels in the liver. Since obese mice (ob/ob) with extensive steatosis also demonstrated decreases in hepatic LRP1 and an increase in hepatic APP, we believe steatosis or liver injury rather than alcohol are directly responsible for the modulation of these key proteins involved in maintaining Aβ homeostasis. Increases in hepatic APP alone may dramatically promote AD pathology, as a recent study demonstrated that overexpression of human APP in the liver increased Aβ levels in the periphery and the brain and promoted AD pathology in WT mice [132]. Alcohol and obesity are both known to cause steatosis in the liver and have also been linked to AD. It may be that the decrease in hepatic LRP1 and increase in hepatic APP associated with steatosis cause the liver to become an important source of Aβ in the periphery and promote AD pathology.

### 6.3. Alcohol Promotion of Peripheral Inflammation

Neuroinflammation plays an important role in the pathogenesis of AD. However, peripheral inflammation has also been suggested to contribute to AD pathology [133,134]. Chronic alcohol use causes hepatocyte injury, inhibits DNA synthesis and regeneration in hepatocytes, and activates Kupffer cells, which secrete TNF-α and other cytokines that promote hepatic and peripheral inflammation. Alcohol may also affect bacteria in the gut, and the gut–liver axis theory of alcoholic liver injury posits that alcohol intake causes gut-dysbiosis and increases portal lipopolysaccharide (LPS), which stimulates Kupffer cells to release cytokines, resulting in liver damage [135,136]. The release of TNF-α is believed to play a central role in alcohol-induced liver injury and may be an important mechanism by which alcohol promotes AD. An increase in cerebral TNF-α has been reported in AD [137,138], and our work shows that cerebral TNF-α contributes to AD pathology [139]. Cerebral TNF-α can be centrally produced or be blood-borne, as it is known that peripheral TNF-α can cross the BBB [140,141]. Notably, elevated peripheral TNF-α levels are reported in AD patients, correlate with AD severity, and modulate AD pathology [133,134]. In AD transgenic mice, peripheral TNF-α blockage by the TNFR-Fc fusion protein (etanercept) reduces neuroinflammation, BBB disruption, and AD pathology [139]. Taken together, hepatic and peripheral inflammation, particularly TNF-α release induced by alcohol, may promote AD.

## 7. Conclusions

The current paper reviews published experimental studies, either in cell culture systems using various cell lines that overexpress human APP, BACE-1, and tau proteins or conducted in various AD animal models. A review of the literature highlights significant heterogeneity in the outcomes of the experimental studies, which can be attributed to different alcohol concentrations and the resultant liver injury, duration of alcohol consumption, sex, age at initiation of treatment, and/or the rodent model used. The results largely suggest that chronic alcohol exposure at levels that cause liver injury, seen with moderate alcohol intake in humans, aggravates and promotes AD pathology. A few studies investigating chronic alcohol intake at lower doses also suggest that it may have some protective effects on AD pathology, but further experiments are needed. The mechanisms and pathways involved in the CNS effects of alcohol and their effects on AD pathology are more widely studied and include Toll-like receptor activation, Akt/mTOR pathway, CREB phosphorylation pathway, GSK3β and CDK5 activation, IGF receptor, Aβ generation, microglial activation, and BBB dysfunction. The effects of alcohol on the periphery, and the subsequent impact on AD pathology, although less studied, is a growing area of research. Alcohol may affect peripheral targets, particularly the liver, to disrupt Aβ homeostasis in the periphery and mobilize Aβ to the brain and increase peripheral inflammation to promote AD. Overall, there is prospective space for further preclinical research to gauge the neurobiological mechanisms that underlie the alcohol-mediated effects on AD pathology, which may help identify viable therapeutic targets for alcohol-dependent AD.

## Data Availability

No new data were created nor generated in this manuscript.
